# Morphological change in an isolated population of red squirrels (*Sciurus vulgaris*) in Britain

**DOI:** 10.1098/rsos.240555

**Published:** 2025-01-15

**Authors:** Kim E. Chandler, Eloy Gálvez-López, Stuart Black, Andrew C. Kitchener, Kat Hamill, Bethan Roberts, Sean Doherty, Philip G. Cox

**Affiliations:** ^1^ Hull York Medical School, University of York, York YO10 5DD, UK; ^2^ Centre for Integrative Anatomy, Department of Cell and Developmental Biology, University College London, London WC1E 6BT, UK; ^3^ Department of Geography and Environmental Science, University of Reading, Reading RG6 6EJ, UK; ^4^ Department of Natural Sciences, National Museums Scotland, Edinburgh EH1 1JF, UK; ^5^ School of Geosciences, Institute of Geography, University of Edinburgh, Edinburgh EH8 9XP, UK; ^6^ School of Animal, Rural and Environmental Sciences, Nottingham Trent University, Nottingham NG25 0QF, UK; ^7^ Department of Archaeology and History, University of Exeter, Exeter EX4 4QJ, UK

**Keywords:** skull morphology, Sciuridae, geometric morphometrics, stable isotope analysis, phenotypic plasticity

## Abstract

The mechanical properties of dietary items are known to influence skull morphology, either through evolution or by phenotypic plasticity. Here, we investigated the impact of supplementary feeding of peanuts on the morphology of red squirrels (*Sciurus vulgaris*) from five populations in Britain (North Scotland, Borders, Jersey and two temporally distinct populations from Formby (Merseyside)). Stable isotope analysis confirmed dietary ecology in 58 specimens. Geometric morphometrics were used to analyse three-dimensional and two-dimensional shape variation across 113 crania and 388 mandibles, respectively. Nitrogen isotope ratios (δ^15^N) were lower in the 1990s and 2010s Formby squirrels (suggesting a diet with an increased proportion of peanuts), and higher in other populations. Significant differences in cranio-mandibular shape were found between all populations, with 1990s Formby red squirrels exhibiting a morphology associated with reduced masticatory efficiency. This effect was partially reversed following a reduction in supplementary feeding of peanuts. We propose that these morphological changes are related to the reduced mechanical effort needed to process peanuts relative to naturally occurring food items. This could be an example of diet-induced plastic changes to the skeleton in non-muroid wild mammals, although further research is needed to exclude other driving factors such as genetics.

## 1. Introduction

Understanding the relationship between the anatomy of an organism and the actions it performs—its form and function—is the goal of functional morphological research. One of the most intensively studied systems within this discipline is the morphology of the skull and its interaction with feeding and diet, with examples of craniomandibular variation linked to differences in trophic ecology or food mechanical properties being known across the vertebrates [1–6]. Such morphological variation includes changes to muscle attachment areas and moment arms to increase bite force at certain points along the tooth row, or elongation of the jaw out-levers to enhance jaw-closing velocity [7].

The above examples represent clades in which morphological variation related to diet has accumulated over relatively long time periods. However, much faster evolutionary changes (i.e. changes underpinned by genetics) to craniomandibular morphology in response to changes in available food resources have also been observed. These can result from the introduction of a species to a novel environment, usually an island [8–10], or from ecological changes driven by human activities [11–13]. Phenotypic plasticity is also an important driver of diet-related morphological change. Multiple studies have shown that alterations to the consistency of food provided to laboratory mammals can induce *in vivo* changes to the form of the cranium and mandible through adaptive bone remodelling. Such studies have mostly focused on muroid rodents [14–20], but similar effects have also been recorded in lagomorphs [21], hyraxes [22], carnivorans [23] and primates [24]. Additionally, field studies have suggested that morphological changes seen in rodents introduced to islands may involve plastic responses to novel diets [25,26]. It should also be noted that evolution and plasticity are not necessarily separate phenomena, and that phenotypic plasticity may form an important component of insular evolution, leading over time to heritable change [27,28].

In this study, the relationship between craniomandibular morphology and feeding ecology will be investigated using the case study of red squirrels (*Sciurus vulgaris*) from Great Britain. The population of red squirrels in Britain has suffered a severe decline since the introduction of the invasive eastern grey squirrel (*Sciurus carolinensis*) around 150 years ago [29–32]. Red squirrels have been replaced across much of their original range in Britain, leaving a fragmented distribution of isolated populations [33,34] with low genetic diversity [35]. In mainland Britain, red squirrels are found across most of Scotland except the Central Belt, but in the rest of Great Britain, they are restricted to the northernmost counties (Northumberland and Cumbria) plus some small, isolated populations in Yorkshire, Merseyside and Wales. Additionally, red squirrels are found on some offshore islands including Anglesey, the Isle of Wight and Brownsea Island, and a population is present on Jersey in the Channel Islands [36].

Red squirrels in Great Britain have been isolated from their continental conspecifics since the end of the last glacial period (7–10 ka), and were suggested to have evolved into a separate subspecies (*S. v. leucourus*). However, numerous translocations and re-introductions in more recent times have complicated the picture, leading to the validity of the British subspecies being questioned [37]. For instance, red squirrels almost became extinct in Scotland during the eighteenth century, owing to hunting and a colder climate [31], and the Scottish population was subsequently strengthened with translocations from England and Scandinavia [38]. The Formby population appears to originate from an introduction to Ainsdale, Southport, from Europe (either France or Scandinavia) in the 1930s [33], but little more is known about its origins. Population genetic analyses using nuclear (microsatellite) markers show that the remaining British populations of red squirrels are strongly differentiated, but mitochondrial DNA variation is random and lacks clear geographical structure [34]. Furthermore, the majority of British populations of red squirrels show genetic evidence of continental ancestry, notably including a Scandinavian haplotype that appeared as recently as 1966 that has rapidly become the most dominant in northeastern England [39]. The planting of coniferous forest in Northumberland (Kielder Forest) has connected forest fragments in the north of England with southern Scotland and has resulted in substantial genetic mixing across this region [40]. Further genetic studies have shown that the red squirrel population on Jersey stems from two introductions, one from southern England in the west and one from France in the east, and that this historic genetic footprint has not yet been erased by gene flow across the island [41].

The geographical areas with remaining red squirrel populations provide habitats with differing availability of food items [42]. For example, red squirrels in north Scotland exist almost exclusively on pine seeds (principally Scots pine, *Pinus sylvestris*) [43,44], whereas in Cumbria the mixed forest provides yew seeds (*Taxus baccata*) and hazelnuts (*Corylus avellana*) [45] alongside non-native pine and spruce (*Pinus* spp. and *Picea* spp.). On Jersey, the principal dietary items are acorns from the pedunculate oak (*Quercus robur*) and sweet chestnuts (*Castanea sativa*), although supplementary feeding by members of the public in their gardens is also thought to be important [46]. At Formby, on the Merseyside coast, red squirrels live in a pine wood but received significant year-round supplementary feeding of peanuts (*Arachis hypogaea*) throughout the 1990s and early 2000s from the National Trust and the general public [47]. Supplemental feeding by the National Trust was reduced in response to the detection of squirrelpox in 2007−2009 in an effort to limit disease transmission, and ceased entirely in 2018 following a subsequent outbreak [48]. Members of the public have also been discouraged from feeding the squirrels (M. Frost, 2023, personal communication).

Red squirrels in Britain have been shown to exhibit significant inter-population variation in mandibular morphology [49]. In particular, squirrels from the small population at Formby were notably different in mandibular morphology compared to other populations. This was speculated to be related to differences in diet, with supplementary feeding of peanuts to the squirrels at Formby being proposed to have led to the distinct mandibular shape in this population. However, the research was unable to state definitively that the morphological variation seen in the mandible was driven by diet, nor could it determine whether the change in morphology was the result of evolution over a number of generations, or a product of plastic bone modelling in development or remodelling during adulthood in response to muscle loading within the lifetime of individual squirrels. The study presented here aims to fill the knowledge gaps left by the previous analysis by addressing the following two hypotheses.

**H1**. *Red squirrel specimens collected from Formby during the 1990s will have a distinctive isotopic signal compared to other red squirrel populations in Britain and to more recent Formby squirrels as a result of supplementary feeding*.

This hypothesis is important for understanding whether the dietary differences between populations are substantial enough to potentially drive morphological change. This result is expected owing to the supplementary feeding of peanuts to red squirrels that is known to have taken place at Formby during the 1990s. On average, peanuts represented 25% of dietary items consumed by squirrels at Formby, although this could rise to as high as 57% when pine seed was scarce [47]. Peanuts are legumes and have a lower nitrogen isotope ratio (δ^15^N) than non-leguminous plants by around 3‰ or more [50]. Thus, we predict a similar difference in δ^15^N values in the bone collagen, once trophic enrichment factors are considered between Formby red squirrel specimens from the 1990s, and red squirrels not provisioned with peanuts (such as individuals from other populations in Britain or Formby specimens acquired after supplemental feeding had been reduced). Additionally, we predict less negative carbon isotope ratios (δ^13^C) in the 1990s Formby red squirrels owing to the provision of peanuts. Although peanuts and other squirrel food items all come from C_3_ plants, peanuts are generally grown in warmer climates with increased C_4_ organic matter in the soil. This will produce less negative δ^13^C values compared to nuts and seeds from trees native to Great Britain.

**H2.** *There will be significant morphological differences in both the cranium and mandible between red squirrels from different populations in Britain, and within populations experiencing a significant change in diet*.

This outcome is predicted as the populations under analysis are isolated from one another, with different genetic backgrounds (although without a distinct underlying phylogeographical pattern [34]), and live at different latitudes in different habitats with different climates and available diets (as noted above). Research has suggested that mandibular morphology varies significantly between populations of red squirrels in Britain [37,49], and a link with diet was proposed. It is predicted that similar variation will be seen in cranial morphology, and that differences in craniomandibular morphology will be observed through time in populations where a significant change in the composition of the diet has occurred (as at Formby).

## 2. Material and methods

### 2.1. Sample

**Figure 1.**
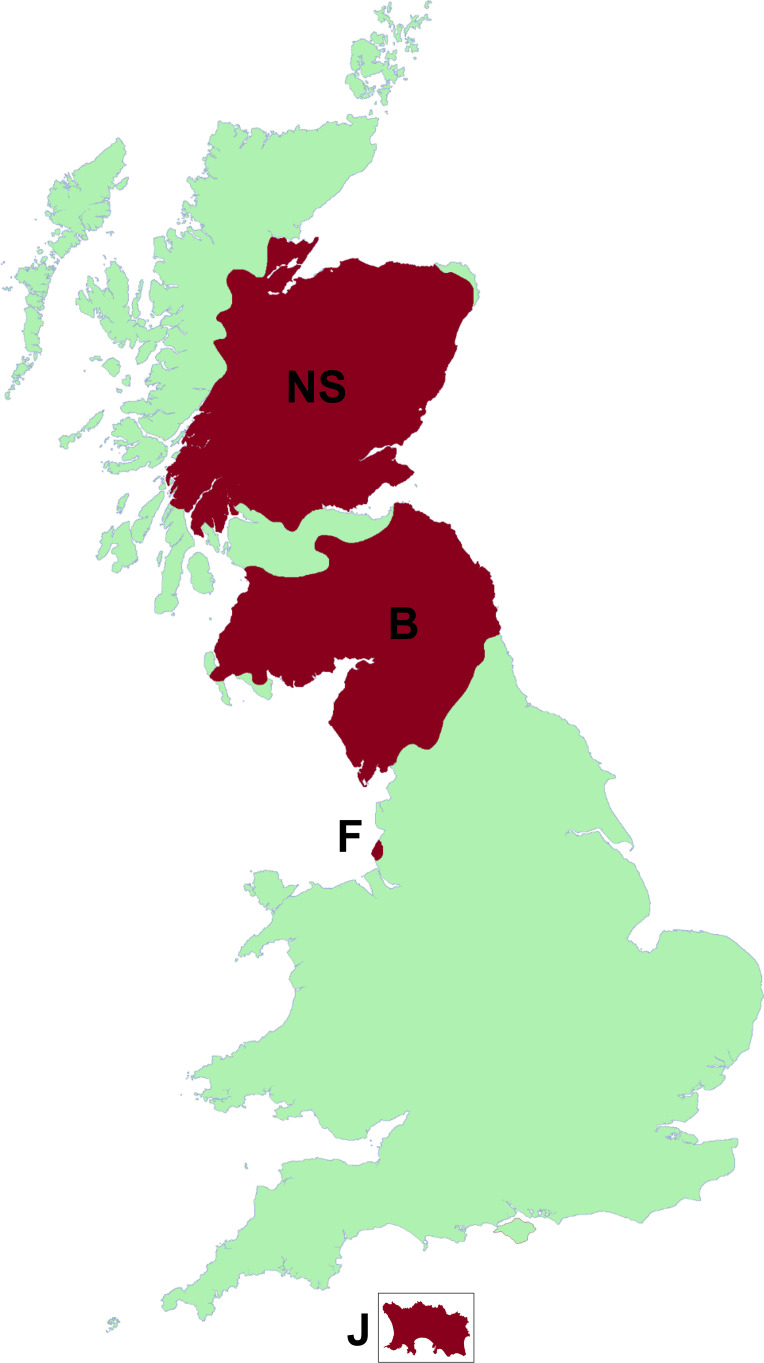
Map showing range of red squirrel populations analysed in this study: B, Borders; F, Formby; J, Jersey (insert, not to scale); NS, North Scotland.

A total of 113 crania and 387 mandibles of red squirrels were obtained from the collections of National Museums Scotland (NMS) for analysis with geometric morphometric methods (GMM). All specimens were opportunistic collections of adult individuals that had died of natural causes or road traffic accidents between 1948 and 2020 in Great Britain and the Channel Islands that were subsequently prepared as osteological specimens by NMS. There are no significant differences between male and female red squirrels in size [51], cranial proportions [52] or, to the best of our knowledge, diet. Nonetheless, our sample had similar numbers of each sex represented within each population in order to minimize any effects of sexual dimorphism (electronic supplementary material, table S1). The specimens were grouped into four groups based on provenance (figure 1): North Scotland (i.e. mainland Scotland north of the Central Belt), the Borders (Scotland south of the Central Belt, plus the English counties of Northumberland, Cumbria and Durham), Formby (the National Trust reserve on the Merseyside coast) and Jersey (Channel Islands). These geographical regions represent areas within which red squirrels can move freely, but between which there is no possibility of populations mixing. Additionally, the specimens from Formby were divided into two groups based on their date of collection: 1990−2000 and 2010−2020. All squirrel specimens from North Scotland, Borders and Jersey were collected before 2011 (and mostly after 1987; electronic supplementary material, table S1). Thus, a total of five populations was analysed in this study. Fewer crania than mandibles were available as many crania were damaged prior to collection (e.g. in road accidents). No intact crania were available for the Jersey specimens. Only one hemi-mandible per specimen was used for GMM. Where both hemimandibles of a specimen were present and intact, the right side was used in preference to the left. Where possible, a rib from each specimen was destructively sampled for stable isotope analysis (57 specimens in total). The number of specimens from each population in each analysis is given in table 1 and the full specimen list is given in the electronic supplementary material, table S1.

**Table 1.** Number of specimens of crania and mandibles (for GMM) and ribs (for stable isotope analysis) in each population.

population	cranium	mandible	rib
North Scotland	27	91	0
Borders	25	89	9
Jersey	0	37	12
Formby (1990−2000)	13	63	18
Formby (2010−2020)	48	105	18
**total**	**113**	**385**	**57**

### 2.2. Stable isotope analysis

Bone collagen extraction from red squirrel ribs was performed at the University of Exeter following the method of Longin [53] with modification from Collins & Galley [54] and Britton *et al*. [55]. Prepared collagen was weighed out in triplicate, and isotope ratio determinations were carried out at the University of Exeter and the University of Reading on a Thermo Fisher DeltaV Plus isotope ratio mass spectrometer coupled to a temperature conversion elemental analyser (TC/EA) with smart EA function. As noted above, δ^15^N and δ^13^C ratios were calculated as both can distinguish between diets high and low in peanuts (see H1 above). δ^15^N and δ^13^C ratios are expressed using the delta notation (δ) in parts per thousand (‰) relative to the international standards of atmospheric N_2_ (AIR) and VPDB (Vienna Pee Dee Belemnite), respectively.

Data were both drift and stretch corrected, and analytical uncertainties were calculated by repeated analyses of internal and international standards. Average uncertainties were ±0.2‰ or less for both elements (2 s.d.). Welch’s ANOVA was used to test for significant differences in isotopic ratios between populations, as this procedure is more robust than the *F*-statistic in unbalanced designs like the present study [56]. Games–Howell post hoc tests were used to test for significant pairwise differences in isotopic ratios between populations.

### 2.3. Geometric morphometrics

**Figure 2.**
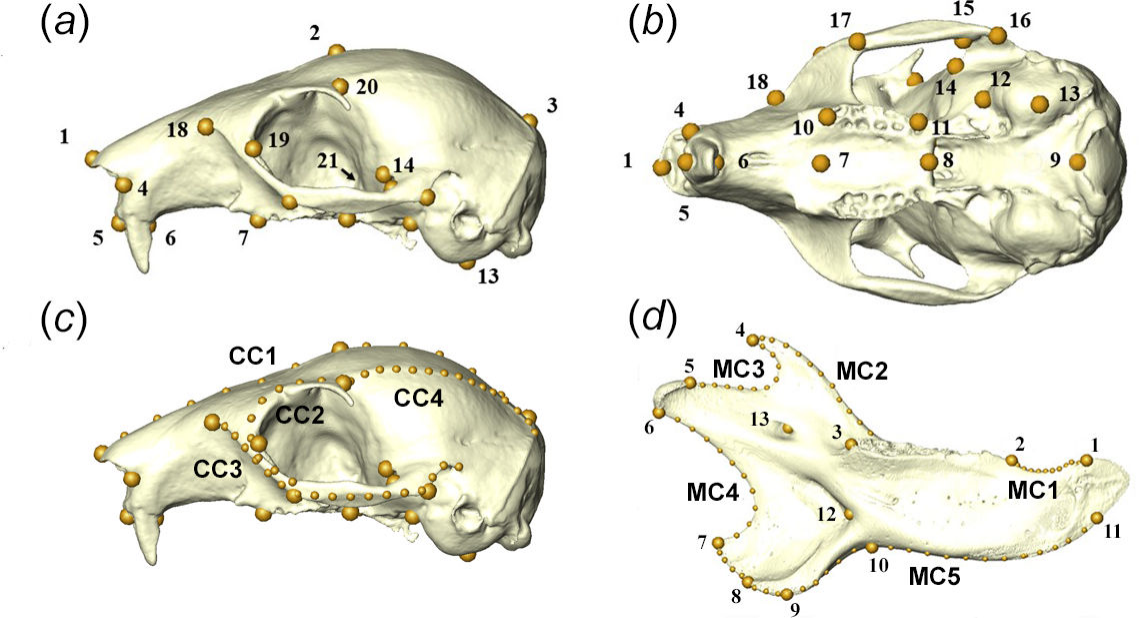
Landmark configurations used for GMM: three-dimensional landmarks (C1−21) shown on red squirrel cranium in (*a*) left lateral and (*b*) ventral view; (*c*) semi-landmarks recorded along four landmark curves (CC1−4); and (*d*) two-dimensional landmarks (M1−13) and semi-landmark curves (MC1−5) shown on labial surface of red squirrel hemi-mandible. Landmark and semi-landmark descriptions are given in table 2.

Red squirrel crania were digitized in three-dimensions using an Artec Space Spider structured-light scanner (Artec3D, Luxembourg). As squirrel hemi-mandibles are largely planar in morphology, two-dimensional photographs of the labial surface were captured with a Nikon D5300 DSLR camera (Nikon Corporation, Tokyo, Japan) with an AF-P DX NIKKOR lens (focal length: 18−55 mm; maximum aperture: f/3.5−5.6G). The camera was secured by a tripod at a constant distance from the bench, with the aperture parallel to the bench to minimize distortions. A set of 21 three-dimensional landmark coordinates was recorded from the cranial meshes using Avizo Lite v. 9.2 (Thermo Fisher Scientific, Waltham, MA, USA) by a single researcher to avoid inter-observer error. Additionally, four curves were digitized representing the dorsal midsagittal line, the anterior margin of the orbit, the zygomatic arch and the superior margin of the temporalis attachment site (hereafter referred to as the ‘temporal ridge’). These curves were converted to equidistantly spaced semi-landmarks using the *Arothron* package [57] in the R statistical environment v. 4.2.3 [58], resulting in 29 cranial semi-landmarks. No subsequent sliding of semi-landmarks was carried out. A set of 13 two-dimensional landmarks on the mandible was recorded using tpsDig2 [59]. Lines were placed along the outline of the hemi-mandible in five sections, excluding the incisor and molar alveoli and the condyle. These lines were then converted to equidistantly spaced semi-landmarks using tpsUtil [59] with no further sliding. This resulted in a total of 82 landmarks and semi-landmarks on the mandible. Landmark positions on both the cranium and mandible are described and illustrated in table 2 and figure 2. Landmark coordinates were aligned using generalized Procrustes superimposition and principal components analysis (PCA) was used to visualize the distribution of squirrel specimens across the shape space. Five specimens were landmarked five times on different days. These repeats were added to the PCA and were shown to cluster closely compared to the overall spread of data indicating good repeatability. Mandibular shape variation along principal components (PC) was visualized using wireframes. To aid visualization of small shape changes in the cranium, mean cranial reconstructions were coloured to represent areas of relative expansion and contraction moving along the first three PCs from negative to positive, using the *localmeshdiff* function of *Arothron* [57]. This function measures the relative change in the area of each facet of the surface mesh and is thus not influenced by the superimposition of the meshes.

**Table 2.** Cranial (three-dimensional) and mandibular (two-dimensional) landmarks and semi-landmarks used in the GMM analyses. (Landmarks and semi-landmarks are visualized in figure 2.)

landmarks		
**cranial landmarks**		
**C1**	anteriormost point on internasal suture	
**C2**	midpoint of line running between dorsal orbital notches	
**C3**	posteriormost point on dorsal midline	
**C4**	anteriormost point on naso-premaxillary suture	
**C5**	anterior midpoint of incisor alveoli	
**C6**	posterior midpoint of incisor alveoli	
**C7**	midpoint between anteriormost points of first premolar alveoli	
**C8**	posteriormost midline point on palate	
**C9**	midline point of the ventral margin of the foramen magnum	
**C10**	anteriormost point on alveolar margin of first premolar	
**C11**	posteriormost point on alveolar margin of third molar	
**C12**	anteriormost point of auditory bulla	
**C13**	ventral apex of auditory bulla	
**C14**	anterior extremity of zygomatic process of squamosal	
**C15**	posteriormost point on anterior margin of zygomatic process of squamosal	
**C16**	posteriormost point on jugal bone	
**C17**	postero-lateral extremity of zygomatic plate	
**C18**	anteriormost extremity of zygomatic plate	
**C19**	ventralmost point of lacrimal tubercule on orbital margin	
**C20**	posterior junction of postorbital process and cranial vault (dorsal orbital notch)	
**C21**	anteriormost point on margin of optic foramen	
**cranial curves**		**no. semi-landmarks**
**CC1**	dorsal midsagittal line of cranium (C1 to C3)	11
**CC2**	anterior orbital margin (C20 to C17)	8
**CC3**	ventral margin of zygomatic arch (from C18 to posterior extremity of zygomatic process of squamosal)	17
**CC4**	temporal ridge from C20 to nuchal line	14
**mandibular landmarks**		
**M1**	dorsalmost point on margin of incisor alveolus	
**M2**	mesial margin of alveolus of first premolar	
**M3**	distal margin of alveolus of third molar	
**M4**	tip of coronoid process	
**M5**	rostral margin of mandibular condyle	
**M6**	caudal margin of mandibular condyle	
**M7**	caudal tip of angular process	
**M8**	ventralmost point of pterygoid fossa on margin of angular process	
**M9**	ventralmost point on margin of angular process	
**M10**	dorsal point of inflection between angular process and mandibular body	
**M11**	ventralmost point on the incisor alveolar margin	
**M12**	rostralmost point of pterygoid fossa	
**M13**	centre of mandibular foramen	
**mandibular curves**		**no. semi-landmarks**
**MC1**	margin of diastema between M1 and M2	10
**MC2**	anterior margin of coronoid process between second molar and M4	10
**MC3**	condylar notch between M4 and M5.	11
**MC4**	posterior margin of mandible between M6 and M7	10
**MC5**	ventral margin of mandible between M7 and M11	28

The following tests were undertaken for both crania and mandibles: (i) Procrustes ANOVA was employed to test for significant morphological differences between populations (i.e. shape ~ population), and post hoc pairwise differences were tested using permutation methods; (ii) Welch’s ANOVA and post hoc Games–Howell tests were used to test for significant differences in centroid size between populations; (iii) multivariate regression was used to test for significant relationships between shape and centroid size in each population; and (iv) differences in the orientation of the allometric trajectory (shape ~ size) of each population were explored with phenotypic trajectory analysis (PTA) [60,61]. Additionally, significant relationships between isotopic ratios and mandibular shape and size were tested with regression methods. Isotopic data were unavailable from sufficient specimens with intact crania to test for relationships between isotopic ratios and cranial shape. Statistical significance was assessed with residual randomization permutation tests of 10 000 repeats (except for Welch’s ANOVA and Games–Howell tests). All analyses and visualizations were carried out using the R packages *Arothron* [57], *effectsize* [62], *geomorph* [63,64], *ggforce* [65], *ggplot2* [66], *Morpho* [67], *RRPP* [68,69], *scales* [70] and *tidyverse* [71].

## 3. Results

### 3.1. Isotopic analysis

**Figure 3.**
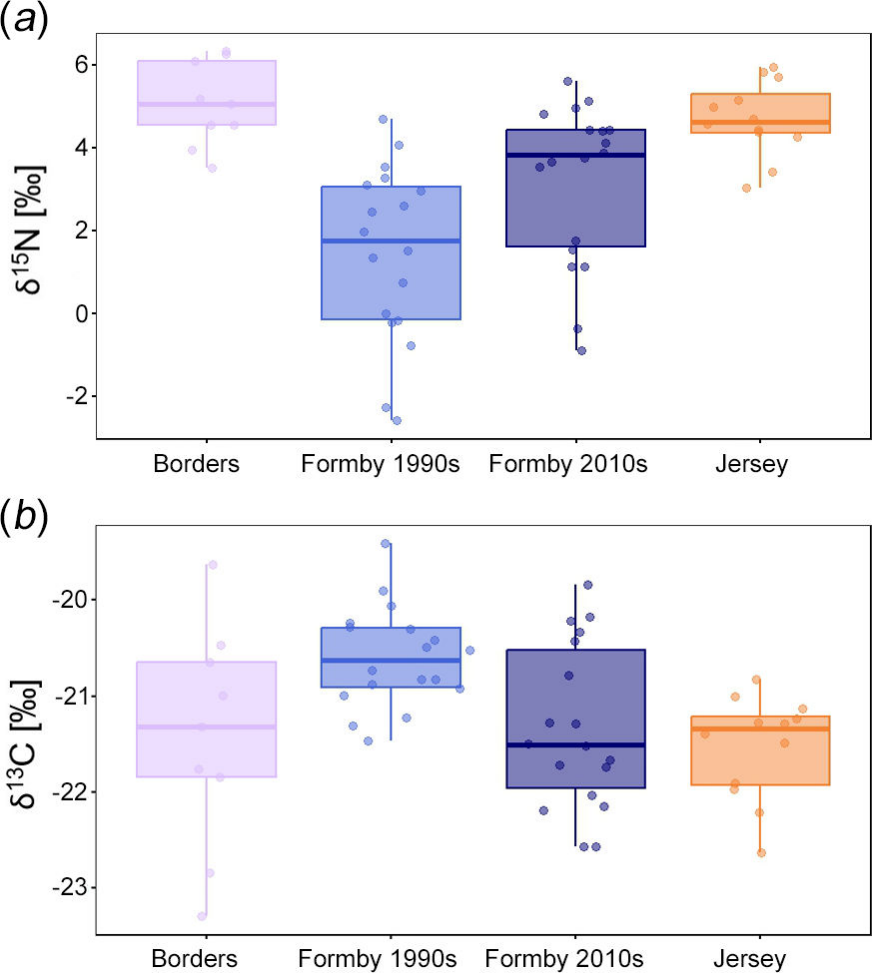
Boxplots showing isotopic ratios (‰) of British red squirrels: (*a*) δ^15^N; and (*b*) δ^13^C. Dots represent data points, bold line shows median and box represents interquartile range. Note that the width of the point scatter is proportional to the density distribution within each group (as in violin plots).

Isotopic values are given in the electronic supplementary material, table S1. ANOVA tests revealed significant differences in δ^15^N values between the populations of red squirrels in this analysis (*F* = 14.43, *p* < 0.001). A post hoc Games–Howell test (for unequal sample sizes and variances) showed significant differences in δ^15^N values between all pairs of populations except Borders and Jersey, and between the two Formby populations (electronic supplementary material, table S2). It can be seen from figure 3*a* that the 1990s Formby population had the lowest mean δ^15^N value, followed by the 2010s Formby squirrels, and that the squirrels from the Borders and Jersey had the highest δ^15^N values. An ANOVA also found significant differences in δ^13^C values (*F* = 8.12, *p* < 0.001), with a post hoc Games–Howell test confirming two pairs of populations with significant differences: 1990s and 2010s Formby; and 1990s Formby and Jersey (electronic supplementary material, table S3). Figure 3*b* shows similar δ^13^C values between the 2010s Formby, Borders and Jersey populations, but less negative values in the 1990s Formby red squirrels.

### 3.2. Cranial morphology

**Figure 4.**
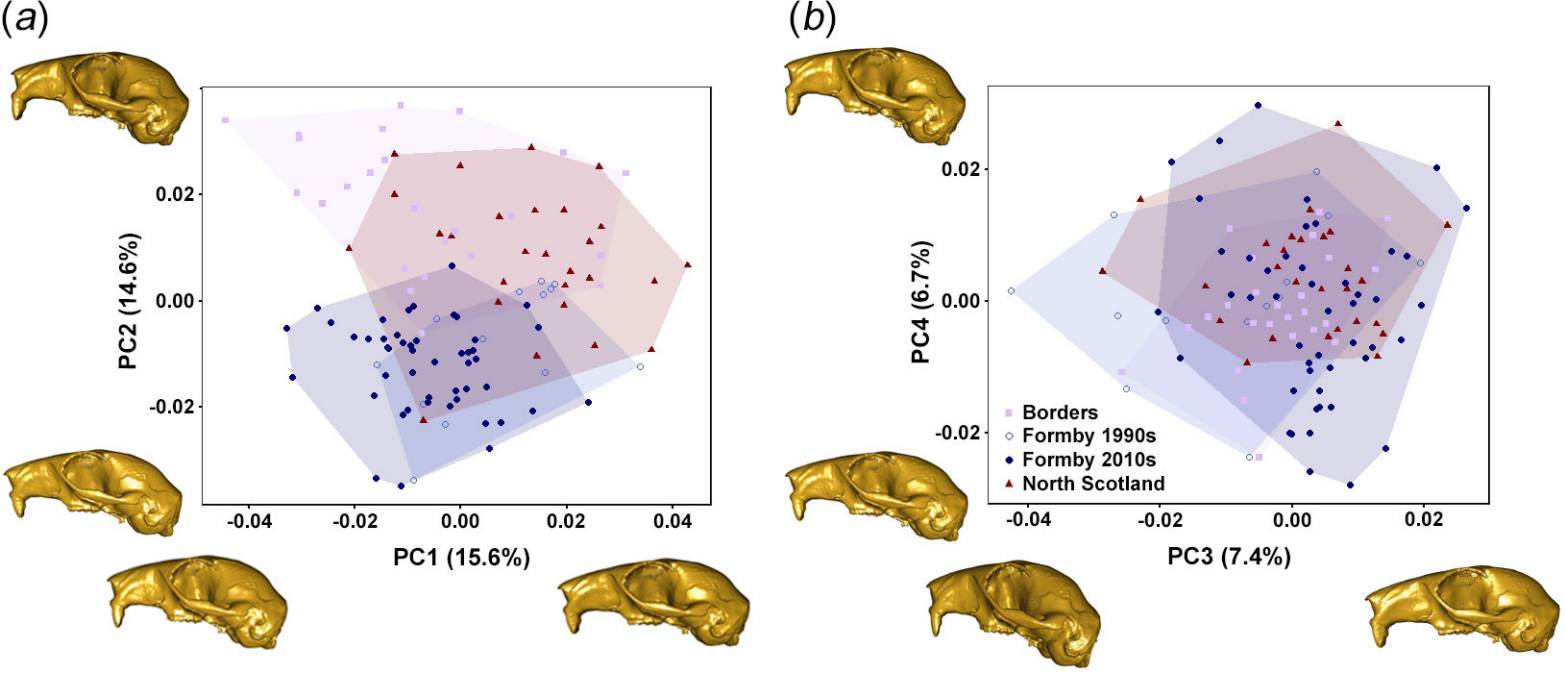
Shape space showing results of GMM analysis of British red squirrel crania: (*a*) PC2 versus PC1; and (*b*) PC4 versus PC3. Surface warps show cranial morphology at five times the minimum and maximum values of each axis in order to increase the visibility of changes.

**Figure 5.**
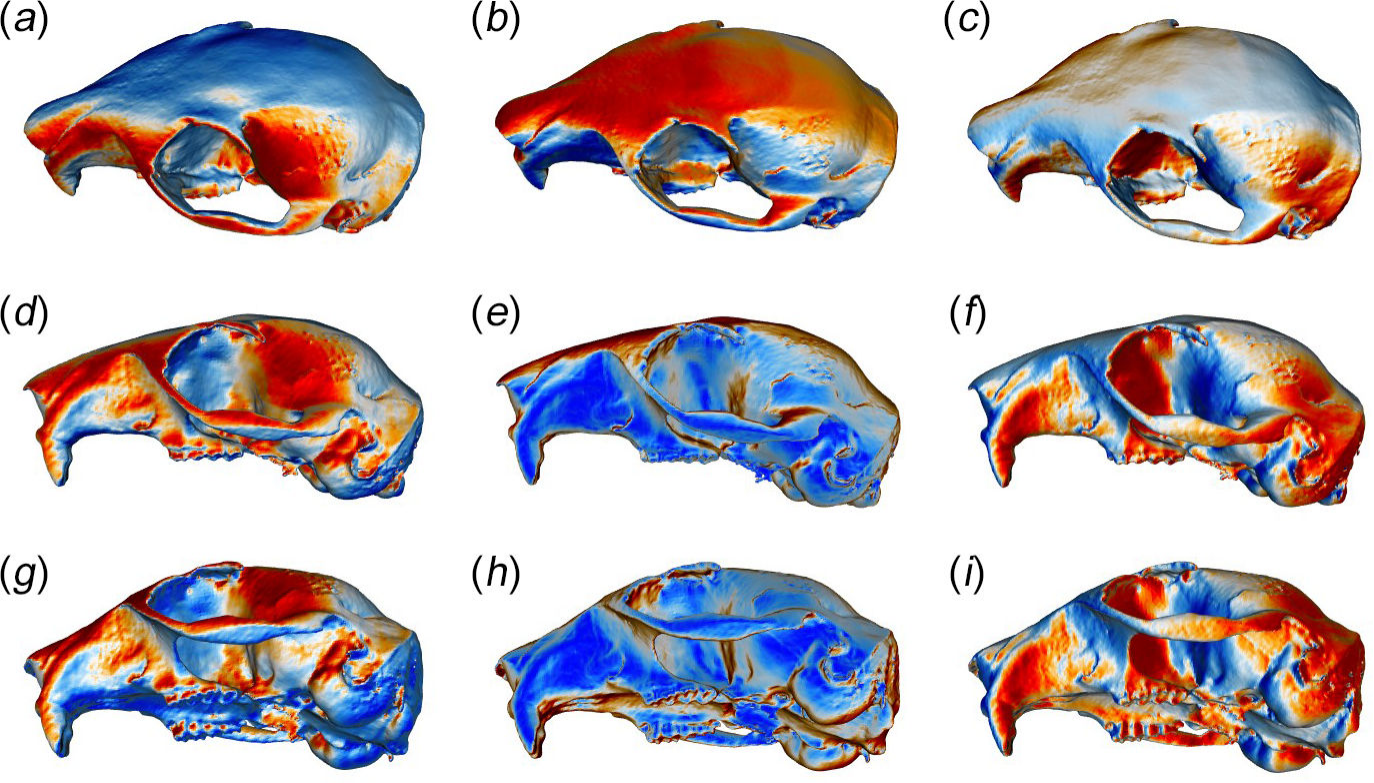
Warped crania in (*a–c*) dorsoventral, (*d–f*) left lateral and (*g–i*) ventrolateral view showing areas of relative expansion (warm colours) and contraction (cool colours) when moving from the minimum to maximum values along (*a,d*) PC1, (*b,e*) PC2, and (*c,f*) PC3.

Figure 4 shows the distribution of red squirrel crania across the first three PCs of the shape space and warped surface reconstructions representing the shape changes along those axes. An alternative method of visualizing shape changes along PCs—colour maps representing areas of relative expansion and contraction—can be shown in figure 5.

PC1 (15.6% variation) represents a trend from a more rounded skull with a ventrally displaced temporal ridge (negative values) to a dorsally flatter, but laterally expanded, cranial vault with a relatively larger orbit, anteriorly expanded nasal region and a more dorsally positioned temporal ridge (positive values). There is considerable overlap between populations on PC1, although some separation can be seen between the older and newer Formby specimens towards the positive and negative ends of the axis respectively. PC2 (14.6% variation) represents a trend from a flatter cranial vault with a ventrally positioned temporal ridge, a laterally expanded zygomatic arch and a longer rostrum (negative values), to a more rounded vault with a dorsally displaced temporal ridge, a medially contracted zygomatic arch and a more contracted rostrum and facial region (positive values). This component separates red squirrels from Formby on the negative half of this axis from the other two populations towards the positive end of the axis. Thus, Formby squirrels have the most flattened cranial vaults of all three populations, with smaller orbits and longer rostra. PC3 (7.4% variation) shows considerable overlap between the populations, similar to PC1. This axis represents a trend towards a more posteriorly expanded but less ventrally deflected occipital region, flatter frontal bones and a less laterally rounded zygomatic arch, moving in a positive direction along the axis. PC4 (6.7% variation) seems to represent the variation seen within the Formby 2010s population, which is spread all along this axis, unlike the other populations. The shape change is mostly an antero-posterior shortening of the skull from negative to positive values.

Although there was substantial overlap between all four populations, significant morphological differences were detected between them (*F* = 6.97, *R*^2^ = 0.161, *p* = 0.001). Post hoc permutation tests found significant differences (*p* < 0.05) between all pairs of populations including between the two cohorts from Formby (electronic supplementary material, table S4). Differences between mean cranial shapes of populations are given in the electronic supplementary material, figure S1, and reflect the shape changes along axes described above. The shape changes between the Formby cohorts are those seen along PC1, and the shape changes from the Formby populations to North Scotland to Borders are those seen along PC2.

**Figure 6.**
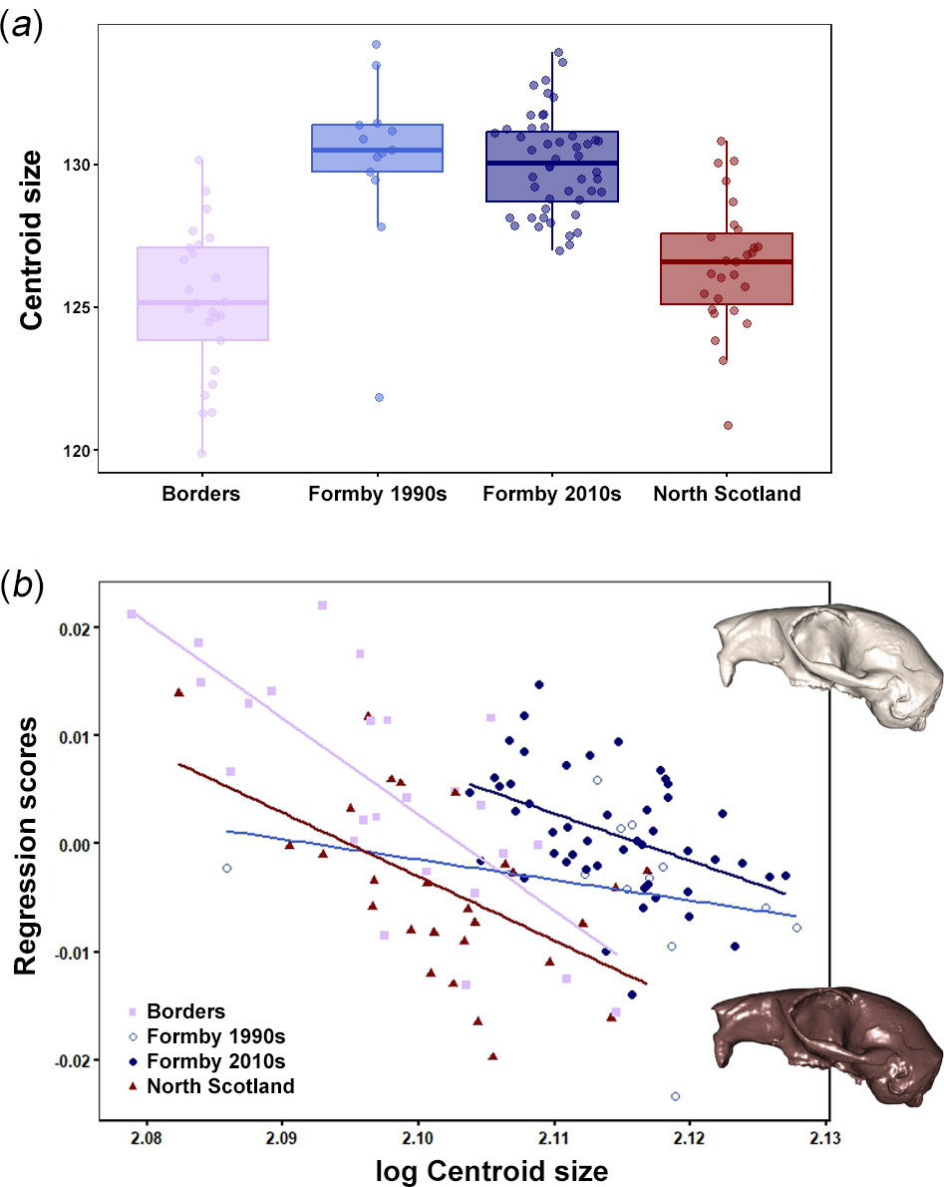
(*a*) Boxplots showing the cranial size of red squirrels from British populations. Dots represent data points, bold line shows median and box represents interquartile range. Note that the width of the point scatter is proportional to the density distribution within each group (as in violin plots). (*b*) PTA of the red squirrel cranium. Surface warps represent cranial shapes at the minimum (light brown) and maximum (dark brown) centroid sizes, magnified five times to increase the visibility of morphological changes.

Cranial size was significantly different between populations (*F* = 34.88, *R*^2^ = 0.490, *p* < 0.001; figure 6*a*), with a post hoc Games–Howell test finding significant differences in cranial size between all pairs of populations except Borders and North Scotland, and the Formby 1990s and 2010s populations (electronic supplementary material, table S5). Significant relationships were found between cranial shape and size in the Borders, North Scotland and Formby 2010s populations but not Formby 1990s (see table 3 for statistics; figure 6*b*). *R*^2^ values were low for all populations (> 0.14) indicating that only a small proportion of cranial shape variation is explained by size variation. PTA was used to compare the trajectories of pairs of populations (table 4). Significant differences in orientation were found between Formby 1990s and all other populations (with angles of over 70° between trajectories). There was no evidence for significant differences in trajectory orientation between the remaining pairs of red squirrel populations, although angles between trajectories were still relatively large (50°−60°).

**Table 3.** Results of multivariate regression of cranial shape on cranial size in each red squirrel population.

population	*n*	*R^2^*	*F*	*p*
**Borders**	25	0.136	3.62	<0.001
**Formby 1990s**	13	0.075	0.89	0.619
**Formby 2010s**	48	0.052	2.53	<0.001
**North Scotland**	27	0.096	2.67	0.002

**Table 4.** Results of PTA of cranial allometry in pairs of red squirrel populations.

pairwise comparison	angle	*R^2^*	*Z*	*p*
**Borders:Formby 1990s**	76.25	0.056	2.21	0.013
**Borders:Formby 2010s**	56.93	0.298	0.80	0.213
**Borders:North Scotland**	51.28	0.391	0.07	0.471
**Formby 1990s:Formby 2010s**	70.68	0.109	1.67	0.047
**Formby 1990s:North Scotland**	73.35	0.082	1.78	0.041
**Formby 2010s:North Scotland**	59.97	0.250	0.88	0.190

### 3.3. Mandibular morphology

**Figure 7.**
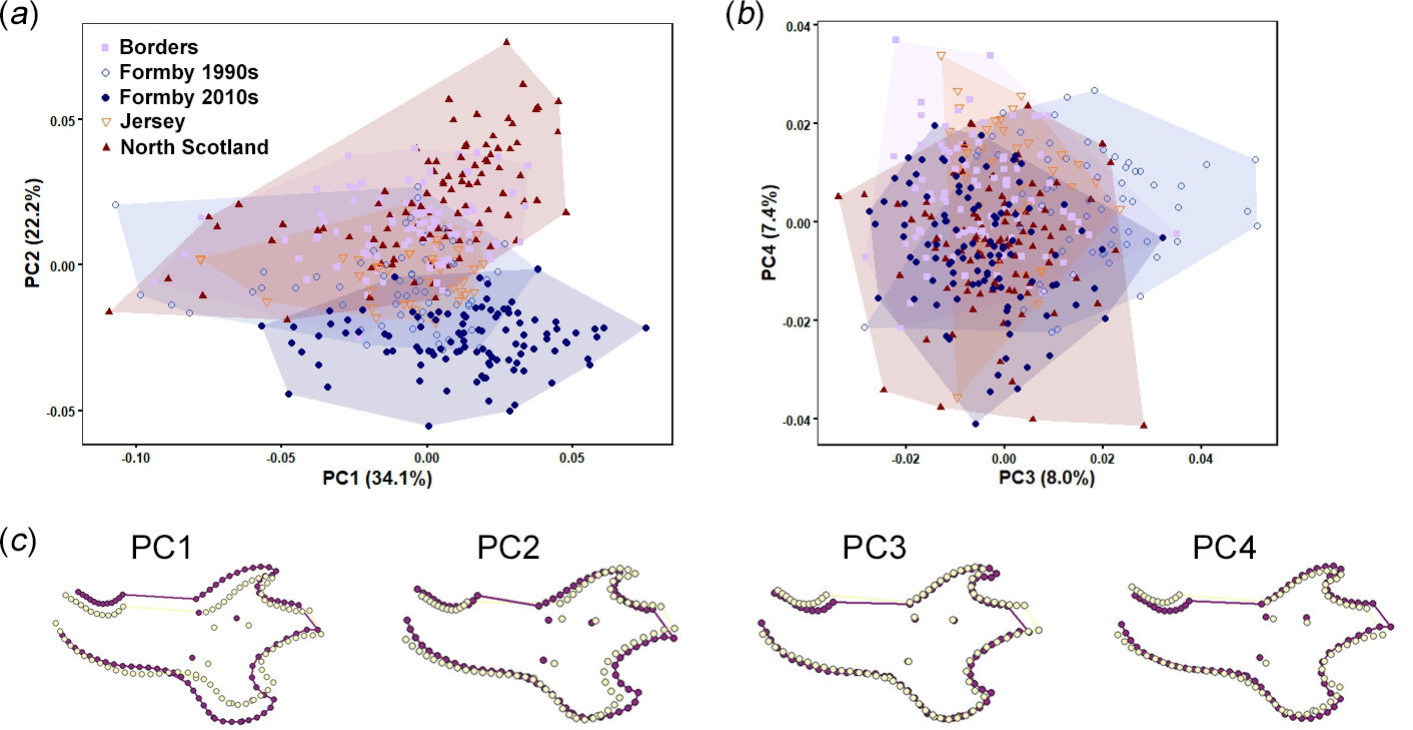
Shape space showing results of GMM analysis of British red squirrel mandibles: (*a*) PC2 versus PC1; and (*b*) PC4 versus PC3. Warped wireframes of red squirrel mandibles in left lateral view show morphology at the minimum (yellow) and maximum (purple) extremes of each axis.

The distribution of red squirrel mandibles across the first three PCs of the shape space resulting from the GMM analysis is shown in figure 6 along with wireframes showing shape changes along each component between the negative and positive extremes. PC1 (34.1% variation) represents a deepening of the mandible relative to its length from negative to positive values, alongside a shortening of the diastema and a ventral expansion of the angular process. From negative to positive values along PC2 (22.2% variation) the molar toothrow shortens relative to mandibular length, the coronoid process becomes more vertically oriented and wider, and the angular process becomes broader and more posteriorly directed. Although there is considerable overlap, the four geographical populations show some separation along PC2, with Formby squirrels having the most negative values, followed by Jersey, the Borders region and finally North Scotland with the most positive PC2 values. Within the Formby squirrels, the more recent (2010s) specimens have more negative PC2 values than the older (1990s) specimens, although there is some overlap. Shape variation along PC3 (8.0% variation) is principally concentrated on the coronoid process, representing a change from a blunt morphology at the negative end of the axis to a highly curved process at the positive extreme. This axis discriminates the Formby squirrels collected in the 1990s (positive values) from those collected more recently in the 2010s, which group together with Borders and North Scotland squirrels (negative values) (figure 7). Jersey squirrels occupy the intermediate region between those clusters. PC4 (7.4% variation) shows little separation of the populations and represents the diastema becoming slightly shallower and a posterior shift of the condyle moving from negative to positive along the axis.

**Figure 8.**
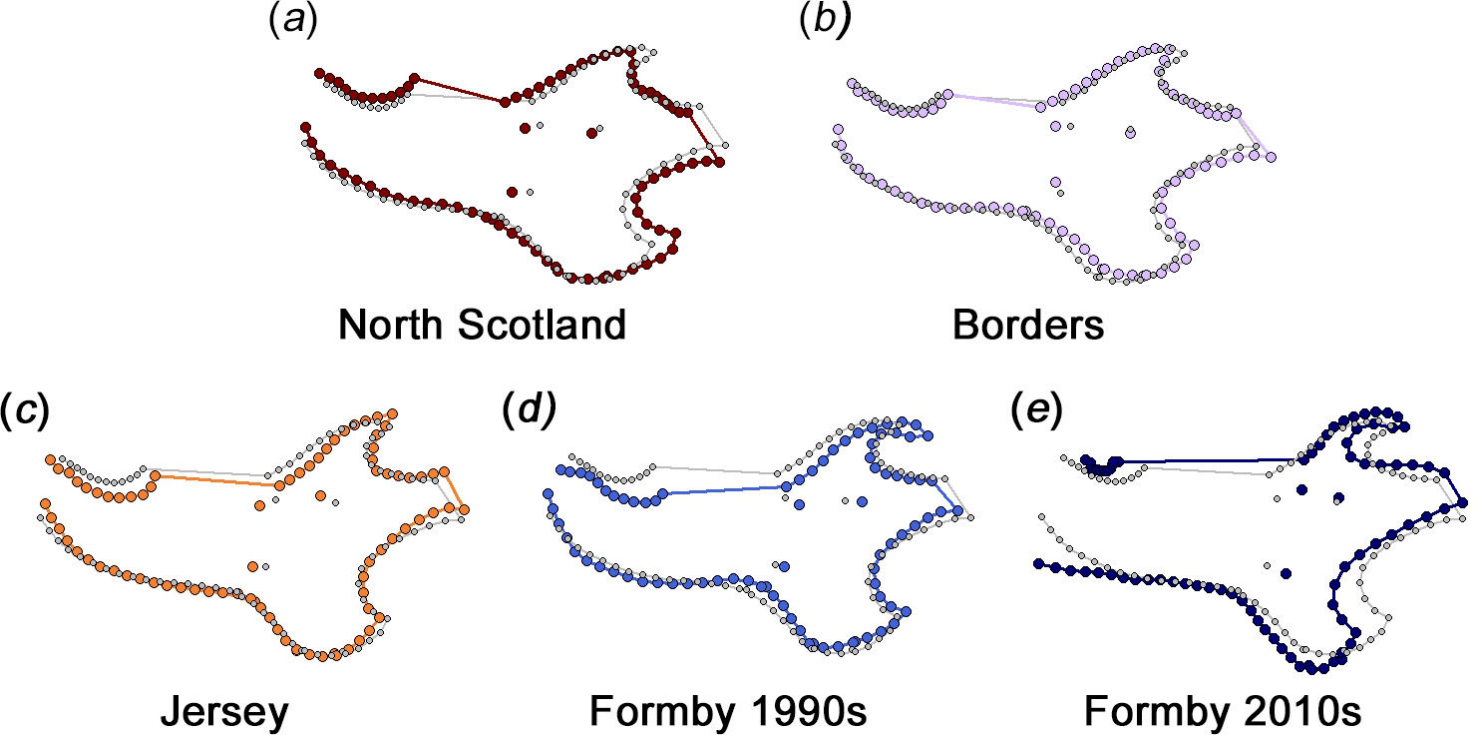
Mean mandibular morphologies: (*a*) North Scotland; (*b*) Borders; (*c*) Jersey; (*d*) Formby 1990s; and (*e*) Formby 2010s. Smaller, grey points show the total sample mean shape. Larger, coloured points represent the mean shapes of red squirrel populations, magnified five times to increase the visibility of shape changes. Unmagnified mean shapes are given in the electronic supplementary material, figure S2.

Significant mandibular shape variation was found between the red squirrel populations (*F* = 28.74, *R*^2^ = 0.231; *p* = 0.001). Permutation tests found significant differences in mandibular morphology (*p* < 0.05) between all pairs of populations (electronic supplementary material, table S6). The mean mandibular shape of each population is shown relative to the overall sample mean shape in figure 8 (shape changes magnified five times) and the electronic supplementary material, figure S2 (unmagnified shape changes). It can be seen that the Jersey and 1990s Formby mean mandibles are more gracile, whereas the other populations have deeper, more robust mandibular bodies. Both Formby populations show posteriorly extended coronoid processes, as does Jersey although to a slightly lesser extent. The North Scotland and Borders mean mandibles have more dorsally directed coronoid processes. The 2010s Formby mean mandible has a more ventrally deflected angular processes than that of the other populations.

**Figure 9.**
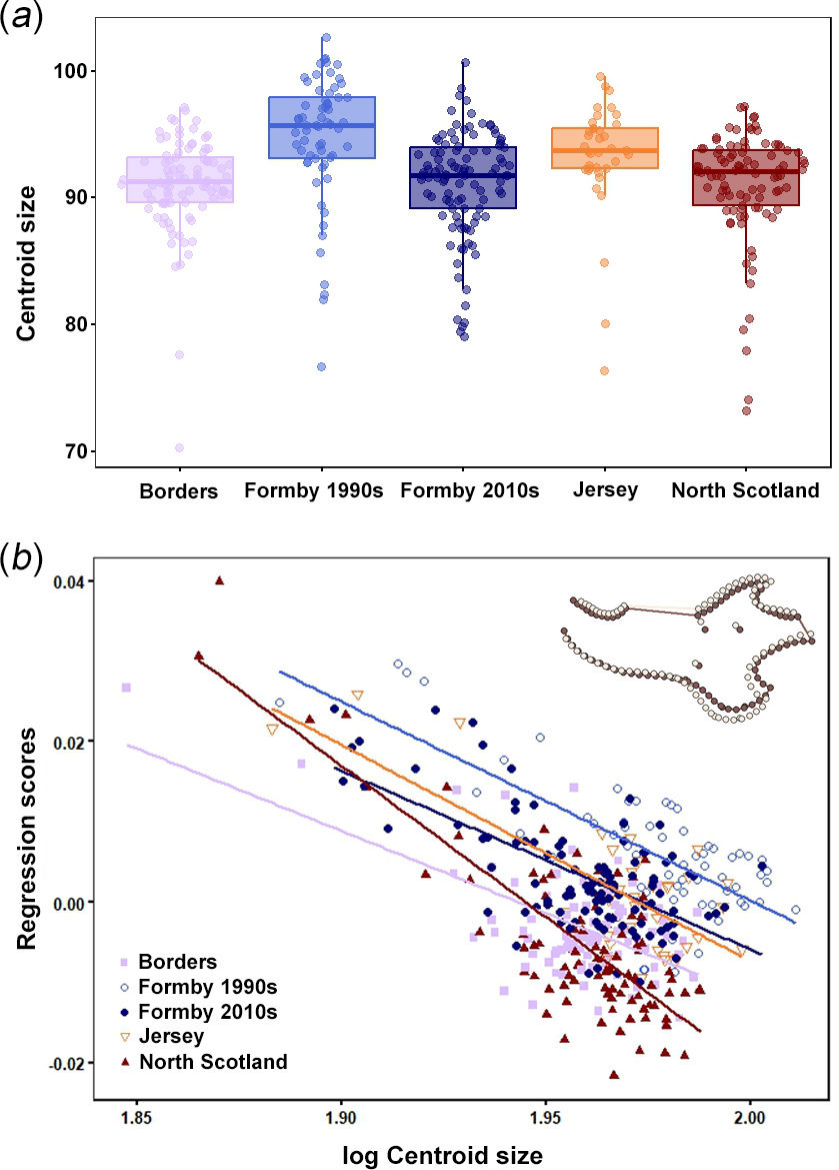
(*a*) Boxplots showing the mandibular size of red squirrels from British populations. Dots represent data points, bold line shows median and box represents interquartile range. Note that the width of the point scatter is proportional to the density distribution within each group (as in violin plots). (*b*) PTA of the red squirrel mandible. Outlines represent mandibular shapes at allometric extremes: light brown, regression score for smallest centroid size; dark brown, regression score for largest centroid size.

Mandibular size differences between populations were detected (*F* = 10.40, *R*^2^ = 0.098, *p* = 0.001; figure 9*a*), but they were driven by red squirrels from the 1990s of Formby being significantly larger than all other populations and time periods except for those from Jersey (electronic supplementary material, table S7). A significant relationship between mandibular shape and size was found in all red squirrel populations (table 5 and figure 9*b*). *R*^2^ values were larger (0.151−0.311) than in the cranial allometric analysis, showing that a greater proportion of shape variation is explained by size variation in mandibles than in crania.

**Table 5.** Results of multivariate regression of mandibular shape on mandibular size in each red squirrel population.

population	*n*	*R^2^*	*F*	*p*
**Borders**	90	0.151	15.51	<0.001
**Formby 1990s**	63	0.311	27.47	<0.001
**Formby 2010s**	105	0.197	25.26	<0.001
**Jersey**	37	0.213	9.49	<0.001
**North Scotland**	93	0.283	35.85	<0.001

PTAs revealed a complex pattern of intraspecific allometry in the mandibles of red squirrels in Britain (statistics in table 6). Significant differences in allometric trajectory orientation were found between Borders and Formby 2010s, Borders and Jersey, North Scotland and Jersey, North Scotland and Formby 1990s, and North Scotland and Formby 2010s, with angles between trajectories ranging between 20.9° and 33.8°. No evidence of differences in orientation were found between the remaining pairs of populations (range of angles: 18.4°−22.8°).

**Table 6.** Results of PTA of mandibular allometry in pairs of red squirrel populations.

pairwise comparison	angle	*R^2^*	*Z*	*p*
**Borders:Formby 1990s**	18.35	0.901	0.92	0.180
**Borders:Formby 2010s**	20.91	0.873	1.69	0.045
**Borders:Jersey**	27.48	0.787	1.74	0.041
**Borders:North Scotland**	20.09	0.882	1.46	0.073
**Formby 1990s:Formby 2010s**	18.73	0.897	1.48	0.068
**Formby 1990s:Jersey**	22.78	0.850	1.12	0.132
**Formby 1990s:North Scotland**	28.65	0.770	3.37	>0.001
**Formby 2010s:Jersey**	19.92	0.884	0.65	0.257
**Formby 2010s:North Scotland**	23.00	0.847	2.53	0.005
**Jersey:North Scotland**	33.81	0.690	2.73	0.004

**Figure 10.**
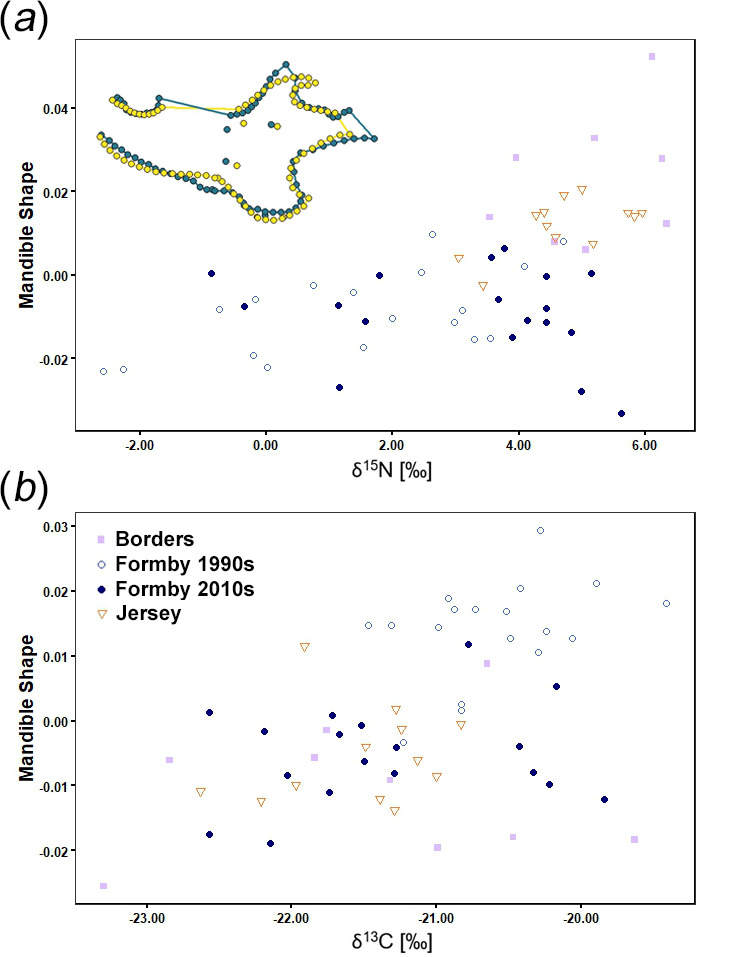
Multivariate regression plots of mandibular shape versus stable isotope ratios (‰): (*a*) δ^15^N; and (*b*) δ^13^C. Warped wireframes of red squirrel mandibles in left lateral view show morphology at the minimum (yellow) and maximum (purple) extremes of the mandibular shape axis in (*a*), magnified five times to increase the visibility of shape changes.

Multivariate regression shows a significant relationship (*F* = 2.44, *R*^2^ = 0.043, *p* = 0.030) between mandibular shape and δ^15^N values (figure 10*a*). Low δ^15^N values are correlated with a more posteriorly curved coronoid process and an anteriorly shifted condyle, compared to mandibles with high δ^15^N values. There is no significant relationship between mandibular morphology and δ^13^C values (*F* = 1.01, *R*^2^ = 0.018, *p* = 0.384; figure 10*b*) and thus the shape change along the regression axis is not explored. No significant relationship was found between mandibular centroid size and either δ^15^N or δ^13^C values (respectively: *F* = 0.77, *R*^2^ = 0.014, *p* = 0.377; *F* = 0.63, *R*^2^ = 0.011, *p* = 0.436).

## 4. Discussion

In this study, we aimed to uncover the link between craniomandibular morphology and diet using a case study of red squirrel populations in Britain. The isotopic results showed, despite some overlap, some differences in δ^15^N and δ^13^C ratios between the populations, particularly between Formby 1990s and other populations. This appears to be in line with the known diets of red squirrels in Britain, especially the high proportion of peanuts provided at Formby. It was shown, using geometric morphometrics, that the morphologies of both the cranium and the mandible vary significantly between populations from North Scotland, the Scottish-English border region, Formby and Jersey. Additionally, at Formby craniomandibular morphology varies across time between specimens from the 1990s and those from the 2010s.

The nitrogen isotope analysis showed a statistical difference in δ^15^N values between red squirrels from Formby, and those from the Borders region and Jersey, supporting H1. The lower δ^15^N values found in the 1990s Formby red squirrels are probably owing to the high proportion of peanuts in their diet (up to 57% [47]), which were provided as supplementary feeding. Leguminous plants, such as peanuts, fix nitrogen directly from the atmosphere and therefore have a lower ^15^N/^14^N ratio than non-legumes [50], which is reflected in the bone collagen of consumers. In no other population did supplementary feeding occur on such a scale, with red squirrel diets mainly comprising non-leguminous plant seeds, such as pine seeds, hazelnuts, sweet chestnuts, beechnuts and/or acorns, depending on the location [42]. Thus, squirrels from the Borders region and Jersey both showed higher δ^15^N values. Red squirrels collected from Formby between 2010 and 2020 had δ^15^N values higher than (although not significantly different from) those found in the Formby specimens from the 1990s, but lower than those of the Jersey and Borders populations. This appears to reflect the reduction in supplementary feeding by the National Trust that took place at Formby from around 2008 onwards, but also indicates that peanut feeding did not cease entirely (especially by the general public).

The carbon isotope analysis also showed a significant difference in δ^13^C values between the two Formby populations as well as between the older Formby squirrels and the Jersey squirrels. This was predicted by H1 owing to the increased C_4_ soil organic matter in the hot climates in which peanuts are grown. Additionally, we propose that this result may also be driven by squirrels feeding on processed human foods, which was reported to occur at Formby in the 1990s [72]. If such foods incorporated C_4_ crops (e.g. sugar cane, corn), this would result in less negative δ^13^C values [73]. The lack of significant difference between the 1990s Formby and Borders populations is largely a result of the large variance in δ^13^C in the latter group, which itself is probably driven by variation in water-use efficiency and soil composition across this large geographical area [74].

The results also supported H2, in that significant morphological differences in both the cranium and mandible were found between all red squirrel populations in Britain in this study. In general, the skull morphology seen in squirrels from Formby suggests a masticatory apparatus less able to produce a high bite force, compared to that of other British red squirrels. For instance, the cranial vault is flatter in Formby squirrels and the temporal ridge is situated more ventrally, both of which would reduce the available attachment area for the temporalis muscle (figure 5*b*,*e*). Formby squirrels also show lateral flaring of the zygomatic arch that could reduce the vertical component of the pull of the masseter muscle. As well as hinting at biomechanical differences between the squirrel populations, these results are also consistent with the craniofacial evolutionary allometry (CREA) hypothesis [75–77]. CREA predicts that adults of larger species in a group of closely related mammals will have longer faces and smaller braincases. As the 1990s Formby red squirrels were the largest specimens in our analyses and showed longer rostra and flatter skulls (figure 6), this rule seems to hold true for populations within a species as well.

Similarly, differences in mandibular morphology suggest variation in biomechanical performance between populations along PC2. The squirrels at the positive end of this axis (North Scotland; figure 7) have robust jaws with broad muscle attachment areas (coronoid and angular processes), suggesting the ability to produce high bite forces. Squirrels at the negative end of PC2 (Formby; figure 7) have more gracile jaws with reduced muscle attachment sites, indicating the generation of lower bite forces during feeding, but they also show a relatively elongated molar row that may suggest an increased importance of molar chewing (for processing less mechanically resistant food items) compared to incisor gnawing. Finally, the particular mandibular morphology of 1990s Formby squirrels is evident at the positive end of PC3 (figure 7) whereas the 2010s Formby red squirrels are more similar to the other populations in Britain in their coronoid and angular process morphology, and thus are not separated from them on PC3. The jaw morphology driving the separation of the older Formby specimens from the other British red squirrels is a coronoid process that is more curved and displaced towards the mandibular condyle (figure 7), which will tend to shorten the temporalis moment arm, reducing its mechanical advantage [49,78]. Similarly, the specialization towards molar chewing relative to incisor gnawing is developed further through a slight expansion of the angular process (for increased masseter insertion area). These shape changes are very similar to the morphological variation that correlates with δ^15^N values (figure 10*a*). However, although the 1990s Formby squirrels are significantly larger than those of most of the other populations, there is no correlation between δ^15^N values and mandibular centroid size. This may be an artefact of the reduced sample size in the isotopic analyses and a relatively weakly significant relationship between mandibular shape and δ^15^N values (*p* = 0.03).

There are several potential drivers of the morphological differences found between red squirrel populations in Britain. It could be a result of the genetic drift that has probably occurred between the isolated populations [34]. Founder effect may also play a role, as the history of red squirrels in Great Britain has involved numerous translocations from mainland Europe over the past two centuries. Historical records are unclear, but the Formby population may derive from a European introduction in the 1930s [33] and the Jersey population appears to be the result of two introductions of squirrels to the island, from southern England and from France [41]. It has also been noted that genetic diversity in red squirrel populations in Britain is low compared to the rest of Europe [41,79], increasing the possibility of inbreeding effects, particularly in the smaller populations in our analyses (Formby and Jersey). Alternatively, given the biomechanical implications outlined above and the correlation between nitrogen isotope ratio and mandibular morphology (figure 10*a*), the morphological differences may be related to diet. Specifically, the morphology of the 1990s Formby population could have resulted from a diet that was high in peanuts. Peanuts are less mechanically demanding than most other food items exploited by red squirrels, as their shells can be broken through with a much lower force than that needed to fracture hazelnut shells or to remove the scales from conifer cones. Thus, it is possible that the reduced masticatory effort required to access peanuts altered the loading regime across the skull and mandible, ultimately leading to the 1990s Formby squirrels exhibiting a craniomandibular morphology with reduced masticatory efficiency compared to, say, squirrels from north Scotland, which feed almost exclusively on pine seeds [43,44].

The Jersey red squirrels had some aspects of mandibular morphology similar to that seen in the 1990s Formby sample (a gracile mandibular corpus and posteriorly deflected coronoid process; figure 8), which could suggest some link to diet as squirrels on Jersey are known to be provisioned year-round with supplemental food by the public [46]. As the Jersey squirrels have similar nitrogen isotope ratios to those of the Scottish and Borders red squirrels, the supplementary food is unlikely to be peanuts, but other non-leguminous supplementary food items, such as seeds and pre-shelled nuts, could contribute to a less mechanically demanding diet without altering the nitrogen isotopic ratio. However, as mentioned above, the unique genetic background of the Jersey squirrel population and its low genetic diversity could have played a major role in driving their morphological phenotype.

The 2010s population of red squirrels from Formby shows a morphology that is different not only from other populations elsewhere in Britain, but also from the older sample at Formby. Some morphological traits indicate increased mechanical efficiency (e.g. the coronoid process), and so could be driven by the reduction of supplementary feeding of peanuts and thus increased mechanical resistance of the diet. However, the 2010s Formby squirrels still have a jaw morphology that suggests reduced mechanical performance relative to the North Scotland, Borders and Jersey populations and could be a result of peanut feeding not having ceased altogether, as also indicated by the stable isotope analysis (figure 3*a*). However, there are other factors that could be driving skull and jaw morphology instead of, or as well as, dietary change. For instance, the Formby population suffered a population crash in the wake of a squirrelpox outbreak in 2008 [48]. A population bottleneck can produce large differences in allele frequencies through random drift and selection, leading to phenotypic change. Moreover, the 2010s sample probably had high levels of inbreeding, which can lead to developmental instability and asymmetry in the rodent mandible [80].

The different potential drivers of the morphological changes identified here are difficult to tease apart with the current sample which, owing to the protected status of red squirrels in Great Britain, was assembled over many years through opportunistic collection of dead individuals rather than being perfectly planned for the desired analyses. The relatively short time period over which morphological change has been identified at Formby indicates that if this is a genetic change the evolutionary process has been rapid (red squirrel generation time is approximately 1 year [51]). However, rodents are noted to undergo rapid morphological evolution when introduced to new environments, particularly islands, with novel food resources [9,10,12,13,81–83]. Alternatively, the morphology could be a result of phenotypic plasticity, i.e. bone modelling during development or remodelling during adulthood in response to loading within the lifetime of individual squirrels [84,85]. Indeed, given that peanuts would be available to the squirrels from weaning onwards, this suggests that modelling would be a more important contributor than remodelling to the Formby morphology. Plastic changes to skull and jaw morphology in response to altered food consistency have been induced in laboratory animals multiple times [14–25] and have also been reported as possibly occurring in captive animals in zoos [86–90]. Furthermore, the specific morphological changes identified in Formby red squirrels from the 1990s (reduced muscle moment arms and attachment areas) are very similar to those produced in some of the above laboratory studies on mice [19,25]. To distinguish between these processes would require a different sample of squirrels, e.g. an ontogenetic series of red squirrels from Formby or a set of captive squirrels raised on different diets. Given the protected status of red squirrels in the Great Britain, these analyses are not possible. Moreover, plasticity versus evolution may be a false binary in that plasticity may be a component of evolution [27,28,91], and it is likely that both processes have played a part in the morphological distribution present in our sample.

As final reflections, the results here have an interesting parallel in modern humans, in whom it has long been suggested that the modern soft diet has led to jaw shrinkage, dental overcrowding and malocclusion [92]. Furthermore, it has been noted by Mitchell *et al.* [20] that an understanding of how the mechanical properties of food items can impact skeletal morphology has important ramifications for the care of captive animals, particularly where the goal is reintroduction to the wild. This is particularly relevant in the case of red squirrels in Britain, where releases of captive-bred specimens are being used to bolster and re-establish declining populations [93]. Although the results here cannot rule out drivers of morphological change other than diet, it seems that best practice would be to match the known dietary ecology of wild animals in captive settings and when providing supplementary food to wild individuals.

## Data Availability

Cranial meshes, mandibular photographs and morphometric data are all available from the UCL Research Data Repository [94]. Supplementary material is available online [95].
